# Crystal structure of 1-iodo-3-{[4-(*tert*-butyl­sulfan­yl)phen­yl]ethyn­yl}azulene

**DOI:** 10.1107/S2056989015012542

**Published:** 2015-07-04

**Authors:** Sebastian Förster, Wilhelm Seichter, Edwin Weber

**Affiliations:** a Institut für Organische Chemie, TU Bergakademie Freiberg, Leipziger Strasse 29, D-09596 Freiberg/Sachsen, Germany

**Keywords:** crystal structure, azulene, 1,3-disubstitution, C—H⋯π inter­action, I⋯π contact

## Abstract

The title compound, C_20_H_19_IS, features a 1,3-disubstituted azulene involving an ethynylene elongated 4-(*tert*-butyl­sulfanyl)­phenyl sidearm and an iodine atom as the substituents. The azulene ring system is almost planar (r.m.s. deviation = 0.012 Å) and subtends a dihedral angle of 35.7 (1)° with the benzene ring. As a result of the inherent dipole character of the azulene core, a supra­molecular π–π dimer [separation between the centroids of the five- and seven-membered rings = 3.7632 (10) Å] with anti­parallel orientated mol­ecules can be observed in the crystal. The packing is consolidated by an unusual I⋯π(acetyl­ene) contact [I⋯*Cg* = 3.34 Å, C—I⋯*Cg* = 173.3°], and a very weak C—H⋯π inter­action is also found in the structure, with the azulene five-membered ring as the acceptor.

## Related literature   

For background to this work, see: Strachota *et al.* (2008 ▸); Xia *et al.* (2014 ▸). For related structures, see: Förster *et al.* (2012 ▸, 2014 ▸). For the synthesis of the starting compounds 1,3-di­iodo­azulene and 1-(*tert*-butyl­sulfan­yl)-4-ethynyl­benzene, see: Wakabayashi *et al.* (1998 ▸); Mayor *et al.* (2003 ▸). For the Sonogashira–Hagihara cross-coupling reaction, see: Sonogashira *et al.* (1975 ▸). For I⋯π contacts, see: Forni *et al.* (2012 ▸). For C—H⋯π inter­actions, see: Nishio *et al.* (2009 ▸).
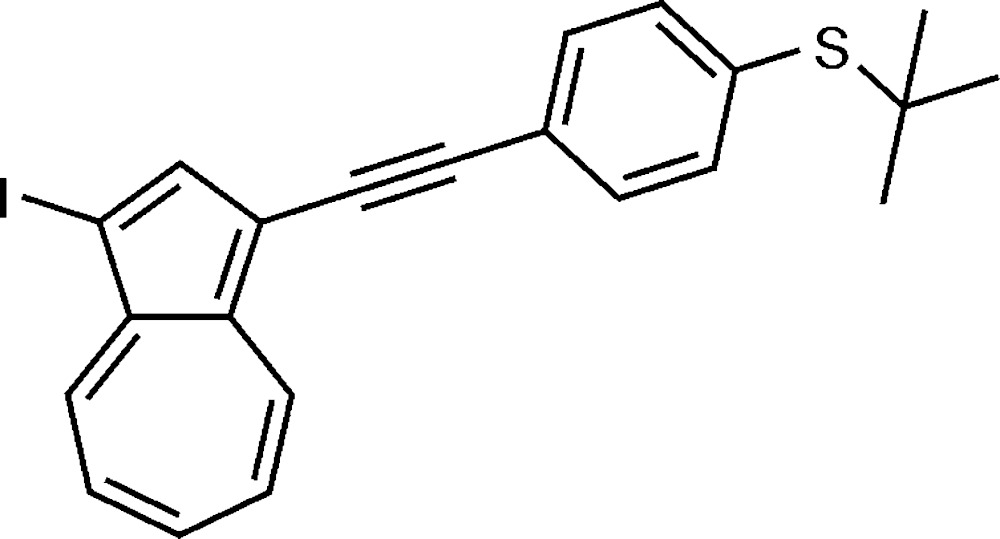


## Experimental   

### Crystal data  

C_22_H_19_IS
*M* *_r_* = 442.33
Monoclinic, 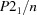

*a* = 12.7222 (2) Å
*b* = 11.9892 (2) Å
*c* = 13.7895 (3) Å
β = 113.851 (1)°
*V* = 1923.68 (6) Å^3^
*Z* = 4
Mo *K*α radiation
μ = 1.77 mm^−1^
*T* = 100 K
0.28 × 0.20 × 0.13 mm

### Data collection  

Bruker Kappa Apex CCD diffractometer
Absorption correction: multi-scan (*SADABS*; Bruker, 2008 ▸) *T*_min_ = 0.637, *T*_max_ = 0.802
23474 measured reflections
5163 independent reflections
4688 reflections with *I* > 2σ(*I*)
*R*_int_ = 0.024

### Refinement  

*R*[*F* ^2^ > 2σ(*F* ^2^)] = 0.021
*wR*(*F* ^2^) = 0.059
*S* = 1.04
5163 reflections
220 parameters
H-atom parameters constrained
Δρ_max_ = 1.37 e Å^−3^
Δρ_min_ = −0.39 e Å^−3^

### 

Data collection: *APEX2* (Bruker, 2010 ▸); cell refinement: *SAINT-NT* (Sheldrick, 2008 ▸); data reduction: *SAINT-NT*; program(s) used to solve structure: *SHELXS97* (Sheldrick, 2008 ▸); program(s) used to refine structure: *SHELXL2013* (Sheldrick, 2015 ▸); molecular graphics: *ORTEP-3 for Windows* (Farrugia, 2012 ▸); software used to prepare material for publication: *SHELXTL* (Sheldrick, 2008 ▸).

## Supplementary Material

Crystal structure: contains datablock(s) I, New_Global_Publ_Block. DOI: 10.1107/S2056989015012542/hb7447sup1.cif

Structure factors: contains datablock(s) I. DOI: 10.1107/S2056989015012542/hb7447Isup2.hkl

Supporting information file. DOI: 10.1107/S2056989015012542/hb7447Isup3.tif

Supporting information file. DOI: 10.1107/S2056989015012542/hb7447Isup4.cml

**Fig. 1.**
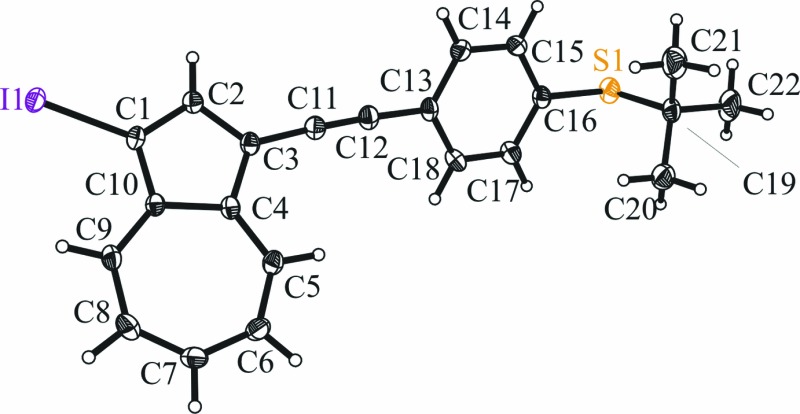
: The molecular structure of the title compound, with displacement ellipsoids drawn at the 50% probability level.

**Fig. 2.**
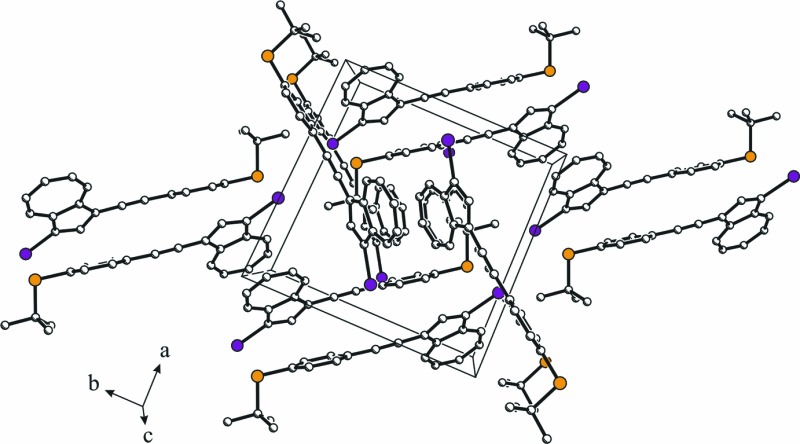
: A view along the c axis of the crystal packing of the title compound.

. DOI: 10.1107/S2056989015012542/hb7447fig1.tif
The mol­ecular structure of the title compound, with displacement ellipsoids drawn at the 50% probability level.

c . DOI: 10.1107/S2056989015012542/hb7447fig2.tif
A view along the *c* axis of the crystal packing of the title compound.

CCDC reference: 1409619

Additional supporting information:  crystallographic information; 3D view; checkCIF report

## Figures and Tables

**Table 1:** Hydrogen-bond geometry (, ) *Cg*1 is the centroid of the C1C4/C10 ring.

*D*H*A*	*D*H	H*A*	*D* *A*	*D*H*A*
C17H17*Cg*1^i^	0.95	2.95	3.471(2)	116

Symmetry code: (i) 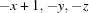.

## supplementary crystallographic information

### S1. Comment

Owing to the remarkable electronic and optical properties of azulene and its derivatives, compounds of this type have recently received enormous inter­est for application in the field of molecular electronics (Strachota *et al.*, 2008; Xia *et al.*, 2014) Being connected to this, the title compound, C_20_H_19_IS, is a valuable inter­mediate in the preparation of corresponding azulenes having a characteristic 1,3-substitution pattern (Förster *et al.*, 2012).

The crystal structure of the compound contains one molecule in the asymmetric part of the unit cell. The molecule deviates from planarity, which can be reflected by a twist angle of 35.7° with respect to the aromatic ring systems of benzene and azulene. The ability of azulene to shift electronic density from the seven-membered ring element toward the five-membered part of the ring system has already been shown to exercise a controlling effect on the supra­molecular inter­actions in the crystal especially regarding π-stacking behavior (Förster *et al.*, 2014). In the present case, supra­molecular dimers with a head-to-tail fashioned orientation of the azulene cores are found (3.44 Å). On the side facing away from the azulene-contact, a benzene ring is located. However, considering the distance and geometry, the existence of a π···π-inter­action can be excluded. Instead, the presence of a weak C—H···π-inter­action [H17···*Cg*(C1, C10) 2.87 Å] is assumed (Nishio *et al.* 2009). In the crystal of the title compound, the packing is furthermore consolidated by a remarkable I···π-inter­action (Forni *et al.* 2012) [I1···*Cg*(C11, C12) 3.34 Å, 173.3°].

### S2.1. Synthesis and crystallization

The title compound was prepared using a Sonogashira–Hagihara cross-coupling reaction (Sonogashira *et al.*, 1975), starting with 1,3-di­iodo­azulene (Wakabayashi *et al.*, 1998) and 1-(*tert*-butyl­sulfanyl)-4-ethynyl­benzene (Mayor *et al.*, 2003). The aryl iodide (8.0 g, 21.06 mmol) and the terminal alkyne (4.0 g, 21.06 mmol) were dissolved in 200 ml of diiso­propyl­amine. After degassing of the solution and cooling to -30 °C, a mixture of bis­(tri­phenyl­phosphane)palladium(II) chloride (110 mg, 0.16 mmol) and copper(I) iodide (60 mg, 0.32 mmol) was added. The mixture was stirred for 18 h at room temperature. After evaporation of the solvent, the residue was purified by column chromatography on SiO_2_ [60 F254 Merck, eluent: hexane] to yield 4.11 g (44 %) of the title compound as a green solid. In addition to the product, 440 mg of 1,3-di­iodo­azulene and 80 mg of 1,3-bis­{[4-(tert-butyl­sulfanyl)phenyl]­ethynyl}azulene were isolated. Analytical data: mp = 120 °C; ^1^H-NMR: (CDCl_3_) *δ*/ppm = 1.32 (s, 9H CH_3_), 7.35 (t, 2H, ArH, ^3^*J*_HH_=9.8 Hz), 7.54 (m, 4H, ArH), 7.72 (d, 1H, ArH, ^3^*J*_HH_=9.9 Hz), 8.10 (s, 1H, ArH), 8.25 (d, 1H, ArH, ^3^*J*_HH_=9.7 Hz), 8.57 (d, 1H, ArH, ^3^*J*_HH_=9.5 Hz); ^13^CNMR: (CDCl_3_) *δ*/ppm = 31.01 (CH_3_), 46.50 (C-(CH_3_)_3_), 74.70 (ArC-I), 86.06 (C≡C), 94.34 (C≡C), 112.17 (ArC), 124.23 (ArC), 125.19 (ArC), 125.61 (ArC), 131.19 (ArC), 132.77 (ArC), 136.15 (ArC), 137.33 (ArC), 139.75 (ArC), 139.77 (ArC), 140.83 (ArC), 141.84 (ArC), 145.77 (ArC); GC/MS calc.: 442; found: 442 [M]+.; EA calc.: C: 59.73 %, H: 4.33 %, S: 7.25 %; found C: 60.03 %, H: 4.35 %, S: 7.21 %; Crystallization by slow solvent evaporation from acetone solution yielded suitable crystals.

### S2.2. Refinement

Crystal data, data collection and structure refinement details are summarized in Table 1. The hydrogen atoms attached to C were fixed geometrically and treated as riding atoms, with d(C—H) = 0.93 and Uiso(H) = 1.2Ueq(C) for aromatic and Uiso(H) = 1.5Ueq(C) for methyl groups.

### Crystal data

**Table:**

C_22_H_19_IS	*F*(000) = 880
*M**_r_* = 442.33	*D*_x_ = 1.527 Mg m^−^^3^
Monoclinic, *P*2_1_/*n*	Mo *K*α radiation, λ = 0.71073 Å
*a* = 12.7222 (2) Å	Cell parameters from 9945 reflections
*b* = 11.9892 (2) Å	θ = 2.5–33.1°
*c* = 13.7895 (3) Å	µ = 1.77 mm^−^^1^
β = 113.851 (1)°	*T* = 100 K
*V* = 1923.68 (6) Å^3^	Irregular, violet
*Z* = 4	0.28 × 0.20 × 0.13 mm

### Data collection

**Table:**

Bruker Kappa Apex CCD diffractometer	4688 reflections with *I* > 2σ(*I*)
phi and ω scans	*R*_int_ = 0.024
Absorption correction: multi-scan (*SADABS*; Bruker, 2008)	θ_max_ = 29.1°, θ_min_ = 1.8°
*T*_min_ = 0.637, *T*_max_ = 0.802	*h* = −17→17
23474 measured reflections	*k* = −16→16
5163 independent reflections	*l* = −18→18

### Refinement

**Table:**

Refinement on *F*^2^	0 restraints
Least-squares matrix: full	Hydrogen site location: inferred from neighbouring sites
*R*[*F*^2^ > 2σ(*F*^2^)] = 0.021	H-atom parameters constrained
*wR*(*F*^2^) = 0.059	*w* = 1/[σ^2^(*F*_o_^2^) + (0.0333*P*)^2^ + 0.9341*P*] where *P* = (*F*_o_^2^ + 2*F*_c_^2^)/3
*S* = 1.04	(Δ/σ)_max_ < 0.001
5163 reflections	Δρ_max_ = 1.37 e Å^−^^3^
220 parameters	Δρ_min_ = −0.39 e Å^−^^3^

### Special details

**Table:**

Geometry. All e.s.d.'s (except the e.s.d. in the dihedral angle between two l.s. planes) are estimated using the full covariance matrix. The cell e.s.d.'s are taken into account individually in the estimation of e.s.d.'s in distances, angles and torsion angles; correlations between e.s.d.'s in cell parameters are only used when they are defined by crystal symmetry. An approximate (isotropic) treatment of cell e.s.d.'s is used for estimating e.s.d.'s involving l.s. planes.

### Fractional atomic coordinates and isotropic or equivalent isotropic displacement parameters (Å^2^)

**Table:**

	*x*	*y*	*z*	*U*_iso_*/*U*_eq_	
I1	0.84013 (2)	0.46782 (2)	0.23899 (2)	0.02206 (5)	
S2	0.11506 (4)	−0.30969 (4)	0.07255 (4)	0.02563 (10)	
C1	0.69313 (13)	0.37992 (13)	0.14949 (13)	0.0173 (3)	
C2	0.64454 (13)	0.29623 (13)	0.18826 (13)	0.0179 (3)	
H2	0.6733	0.2710	0.2596	0.022*	
C3	0.54571 (13)	0.25519 (13)	0.10422 (13)	0.0173 (3)	
C4	0.53172 (13)	0.31415 (13)	0.01096 (13)	0.0163 (3)	
C5	0.44468 (14)	0.29644 (14)	−0.08807 (14)	0.0204 (3)	
H5	0.3916	0.2391	−0.0919	0.024*	
C6	0.42455 (15)	0.35115 (15)	−0.18259 (14)	0.0236 (3)	
H6	0.3600	0.3256	−0.2426	0.028*	
C7	0.48585 (16)	0.43818 (16)	−0.20148 (14)	0.0238 (3)	
H7	0.4551	0.4657	−0.2721	0.029*	
C8	0.58518 (16)	0.49110 (16)	−0.13288 (16)	0.0249 (4)	
H8	0.6140	0.5480	−0.1634	0.030*	
C9	0.64791 (15)	0.47161 (13)	−0.02540 (15)	0.0207 (3)	
H9	0.7142	0.5168	0.0076	0.025*	
C10	0.62711 (13)	0.39541 (13)	0.04023 (13)	0.0160 (3)	
C11	0.47274 (14)	0.16724 (14)	0.10849 (14)	0.0197 (3)	
C12	0.41110 (14)	0.09113 (14)	0.10767 (14)	0.0214 (3)	
C13	0.34086 (13)	−0.00436 (14)	0.10156 (14)	0.0183 (3)	
C14	0.31147 (16)	−0.03408 (14)	0.18573 (15)	0.0230 (4)	
H14	0.3377	0.0096	0.2486	0.028*	
C15	0.24401 (15)	−0.12745 (15)	0.17689 (14)	0.0219 (3)	
H15	0.2249	−0.1478	0.2343	0.026*	
C16	0.20377 (14)	−0.19206 (13)	0.08477 (14)	0.0188 (3)	
C17	0.23511 (15)	−0.16333 (14)	0.00239 (15)	0.0217 (3)	
H17	0.2099	−0.2079	−0.0599	0.026*	
C18	0.30281 (15)	−0.07036 (15)	0.01021 (14)	0.0218 (3)	
H18	0.3234	−0.0514	−0.0467	0.026*	
C19	−0.03091 (15)	−0.24833 (16)	0.01235 (15)	0.0258 (4)	
C20	−0.05345 (17)	−0.19664 (19)	−0.09463 (17)	0.0337 (4)	
H20A	−0.1330	−0.1700	−0.1272	0.051*	
H20B	−0.0411	−0.2527	−0.1407	0.051*	
H20C	−0.0008	−0.1339	−0.0851	0.051*	
C21	−0.04654 (19)	−0.1617 (2)	0.0863 (2)	0.0435 (6)	
H21A	0.0031	−0.0974	0.0917	0.065*	
H21B	−0.0259	−0.1946	0.1567	0.065*	
H21C	−0.1269	−0.1374	0.0579	0.065*	
C22	−0.1104 (2)	−0.3474 (2)	−0.0007 (2)	0.0454 (6)	
H22A	−0.1904	−0.3218	−0.0299	0.068*	
H22B	−0.0920	−0.3824	0.0684	0.068*	
H22C	−0.0999	−0.4018	−0.0491	0.068*	

### Atomic displacement parameters (Å^2^)

**Table:**

	*U*^11^	*U*^22^	*U*^33^	*U*^12^	*U*^13^	*U*^23^
I1	0.01979 (7)	0.02162 (7)	0.02101 (7)	−0.00596 (4)	0.00437 (5)	−0.00162 (4)
S2	0.0270 (2)	0.01496 (19)	0.0329 (2)	−0.00596 (16)	0.01011 (19)	0.00387 (16)
C1	0.0149 (7)	0.0151 (7)	0.0203 (8)	−0.0020 (6)	0.0054 (6)	−0.0022 (6)
C2	0.0170 (7)	0.0161 (7)	0.0202 (8)	0.0007 (6)	0.0070 (6)	0.0020 (6)
C3	0.0165 (7)	0.0136 (7)	0.0214 (8)	−0.0004 (6)	0.0071 (6)	0.0008 (6)
C4	0.0162 (7)	0.0121 (7)	0.0212 (8)	0.0000 (5)	0.0081 (6)	−0.0007 (6)
C5	0.0202 (7)	0.0167 (7)	0.0229 (8)	−0.0031 (6)	0.0073 (7)	−0.0020 (6)
C6	0.0238 (8)	0.0243 (9)	0.0183 (8)	−0.0017 (7)	0.0040 (7)	−0.0010 (6)
C7	0.0294 (9)	0.0243 (8)	0.0176 (8)	0.0005 (7)	0.0094 (7)	0.0021 (6)
C8	0.0269 (9)	0.0213 (8)	0.0295 (10)	−0.0012 (7)	0.0146 (8)	0.0059 (7)
C9	0.0208 (8)	0.0174 (8)	0.0251 (9)	−0.0047 (6)	0.0106 (7)	−0.0014 (6)
C10	0.0157 (7)	0.0135 (7)	0.0193 (7)	−0.0006 (5)	0.0076 (6)	−0.0016 (6)
C11	0.0178 (7)	0.0171 (7)	0.0225 (8)	0.0009 (6)	0.0064 (6)	0.0035 (6)
C12	0.0168 (7)	0.0203 (8)	0.0244 (8)	0.0003 (6)	0.0057 (6)	0.0055 (6)
C13	0.0141 (7)	0.0153 (7)	0.0239 (8)	−0.0005 (6)	0.0060 (6)	0.0037 (6)
C14	0.0229 (8)	0.0243 (9)	0.0195 (8)	−0.0065 (6)	0.0063 (7)	−0.0008 (6)
C15	0.0231 (8)	0.0238 (8)	0.0190 (8)	−0.0051 (6)	0.0087 (7)	0.0018 (6)
C16	0.0181 (7)	0.0140 (7)	0.0240 (8)	−0.0013 (6)	0.0080 (6)	0.0026 (6)
C17	0.0248 (8)	0.0175 (8)	0.0269 (9)	−0.0028 (6)	0.0147 (7)	−0.0041 (6)
C18	0.0240 (8)	0.0207 (8)	0.0261 (9)	−0.0010 (6)	0.0157 (7)	0.0022 (7)
C19	0.0210 (8)	0.0274 (9)	0.0279 (9)	−0.0097 (7)	0.0087 (7)	0.0018 (7)
C20	0.0284 (9)	0.0367 (11)	0.0314 (10)	−0.0069 (8)	0.0072 (8)	0.0088 (8)
C21	0.0270 (10)	0.0586 (15)	0.0486 (14)	−0.0018 (10)	0.0191 (10)	−0.0132 (11)
C22	0.0338 (11)	0.0454 (13)	0.0482 (14)	−0.0219 (10)	0.0073 (10)	0.0110 (11)

### Geometric parameters (Å, º)

**Table:**

I1—C1	2.0651 (15)	C13—C18	1.398 (3)
S2—C16	1.7712 (16)	C13—C14	1.402 (2)
S2—C19	1.8526 (19)	C14—C15	1.386 (2)
C1—C2	1.394 (2)	C14—H14	0.9500
C1—C10	1.409 (2)	C15—C16	1.396 (2)
C2—C3	1.410 (2)	C15—H15	0.9500
C2—H2	0.9500	C16—C17	1.391 (2)
C3—C4	1.414 (2)	C17—C18	1.386 (2)
C3—C11	1.422 (2)	C17—H17	0.9500
C4—C5	1.383 (2)	C18—H18	0.9500
C4—C10	1.479 (2)	C19—C20	1.517 (3)
C5—C6	1.387 (2)	C19—C22	1.523 (3)
C5—H5	0.9500	C19—C21	1.523 (3)
C6—C7	1.389 (3)	C20—H20A	0.9800
C6—H6	0.9500	C20—H20B	0.9800
C7—C8	1.388 (3)	C20—H20C	0.9800
C7—H7	0.9500	C21—H21A	0.9800
C8—C9	1.390 (3)	C21—H21B	0.9800
C8—H8	0.9500	C21—H21C	0.9800
C9—C10	1.385 (2)	C22—H22A	0.9800
C9—H9	0.9500	C22—H22B	0.9800
C11—C12	1.200 (2)	C22—H22C	0.9800
C12—C13	1.434 (2)		
			
C16—S2—C19	102.19 (8)	C13—C14—H14	120.1
C2—C1—C10	109.87 (14)	C14—C15—C16	120.96 (16)
C2—C1—I1	124.97 (12)	C14—C15—H15	119.5
C10—C1—I1	125.15 (12)	C16—C15—H15	119.5
C1—C2—C3	108.81 (14)	C17—C16—C15	119.00 (15)
C1—C2—H2	125.6	C17—C16—S2	120.11 (13)
C3—C2—H2	125.6	C15—C16—S2	120.89 (13)
C2—C3—C4	108.39 (14)	C18—C17—C16	120.67 (16)
C2—C3—C11	127.23 (15)	C18—C17—H17	119.7
C4—C3—C11	124.35 (15)	C16—C17—H17	119.7
C5—C4—C3	125.25 (15)	C17—C18—C13	120.22 (16)
C5—C4—C10	127.69 (15)	C17—C18—H18	119.9
C3—C4—C10	107.06 (14)	C13—C18—H18	119.9
C4—C5—C6	128.67 (16)	C20—C19—C22	110.38 (17)
C4—C5—H5	115.7	C20—C19—C21	110.34 (19)
C6—C5—H5	115.7	C22—C19—C21	110.54 (18)
C5—C6—C7	128.83 (17)	C20—C19—S2	111.05 (13)
C5—C6—H6	115.6	C22—C19—S2	103.93 (14)
C7—C6—H6	115.6	C21—C19—S2	110.44 (14)
C8—C7—C6	129.86 (17)	C19—C20—H20A	109.5
C8—C7—H7	115.1	C19—C20—H20B	109.5
C6—C7—H7	115.1	H20A—C20—H20B	109.5
C7—C8—C9	128.44 (17)	C19—C20—H20C	109.5
C7—C8—H8	115.8	H20A—C20—H20C	109.5
C9—C8—H8	115.8	H20B—C20—H20C	109.5
C10—C9—C8	128.97 (16)	C19—C21—H21A	109.5
C10—C9—H9	115.5	C19—C21—H21B	109.5
C8—C9—H9	115.5	H21A—C21—H21B	109.5
C9—C10—C1	126.65 (15)	C19—C21—H21C	109.5
C9—C10—C4	127.49 (15)	H21A—C21—H21C	109.5
C1—C10—C4	105.87 (13)	H21B—C21—H21C	109.5
C12—C11—C3	176.91 (19)	C19—C22—H22A	109.5
C11—C12—C13	175.55 (19)	C19—C22—H22B	109.5
C18—C13—C14	119.41 (15)	H22A—C22—H22B	109.5
C18—C13—C12	119.05 (16)	C19—C22—H22C	109.5
C14—C13—C12	121.53 (16)	H22A—C22—H22C	109.5
C15—C14—C13	119.71 (16)	H22B—C22—H22C	109.5
C15—C14—H14	120.1		
			
C10—C1—C2—C3	0.28 (19)	C5—C4—C10—C9	1.0 (3)
I1—C1—C2—C3	−179.64 (11)	C3—C4—C10—C9	−179.53 (16)
C1—C2—C3—C4	−0.09 (18)	C5—C4—C10—C1	−179.21 (16)
C1—C2—C3—C11	177.91 (16)	C3—C4—C10—C1	0.28 (17)
C2—C3—C4—C5	179.39 (15)	C18—C13—C14—C15	−0.8 (3)
C11—C3—C4—C5	1.3 (3)	C12—C13—C14—C15	−179.58 (16)
C2—C3—C4—C10	−0.12 (17)	C13—C14—C15—C16	−0.6 (3)
C11—C3—C4—C10	−178.19 (15)	C14—C15—C16—C17	1.8 (3)
C3—C4—C5—C6	179.33 (17)	C14—C15—C16—S2	−178.67 (14)
C10—C4—C5—C6	−1.3 (3)	C19—S2—C16—C17	−89.74 (15)
C4—C5—C6—C7	−0.8 (3)	C19—S2—C16—C15	90.77 (15)
C5—C6—C7—C8	2.6 (3)	C15—C16—C17—C18	−1.6 (3)
C6—C7—C8—C9	−1.7 (3)	S2—C16—C17—C18	178.85 (14)
C7—C8—C9—C10	−0.4 (3)	C16—C17—C18—C13	0.2 (3)
C8—C9—C10—C1	−179.23 (18)	C14—C13—C18—C17	1.0 (3)
C8—C9—C10—C4	0.5 (3)	C12—C13—C18—C17	179.80 (16)
C2—C1—C10—C9	179.47 (16)	C16—S2—C19—C20	60.73 (15)
I1—C1—C10—C9	−0.6 (2)	C16—S2—C19—C22	179.40 (14)
C2—C1—C10—C4	−0.35 (18)	C16—S2—C19—C21	−62.02 (16)
I1—C1—C10—C4	179.57 (11)		

### Hydrogen-bond geometry (Å, º)

Cg1 is the centroid of the C1–C4/C10 ring.

**Table:**

*D*—H···*A*	*D*—H	H···*A*	*D*···*A*	*D*—H···*A*
C17—H17···*Cg*1^i^	0.95	2.95	3.471 (2)	116

Symmetry code: (i) −*x*+1, −*y*, −*z*.
