# Supplementary figures and images for: Crystal structure of 1-iodo-3-{[4-(tert-butyl­sulfan­yl)phen­yl]ethyn­yl}azulene
Source: Acta Crystallogr E Crystallogr Commun. 2015 Jul 4;71(Pt 8):o544–5. doi: 10.1107/S2056989015012542 (PMC4571388; doi:10.1107/S2056989015012542)

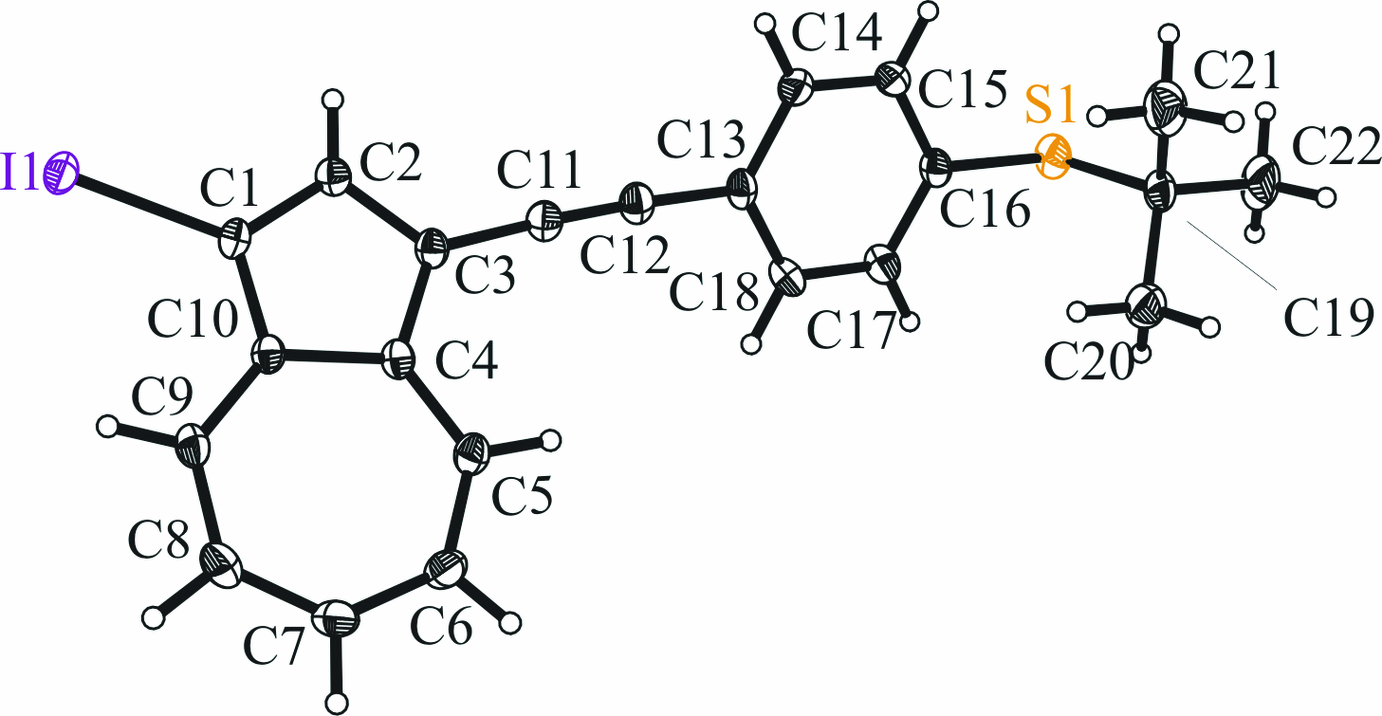

Supplement: Supplementary file 5 [file e-71-0o544-fig1.tif]

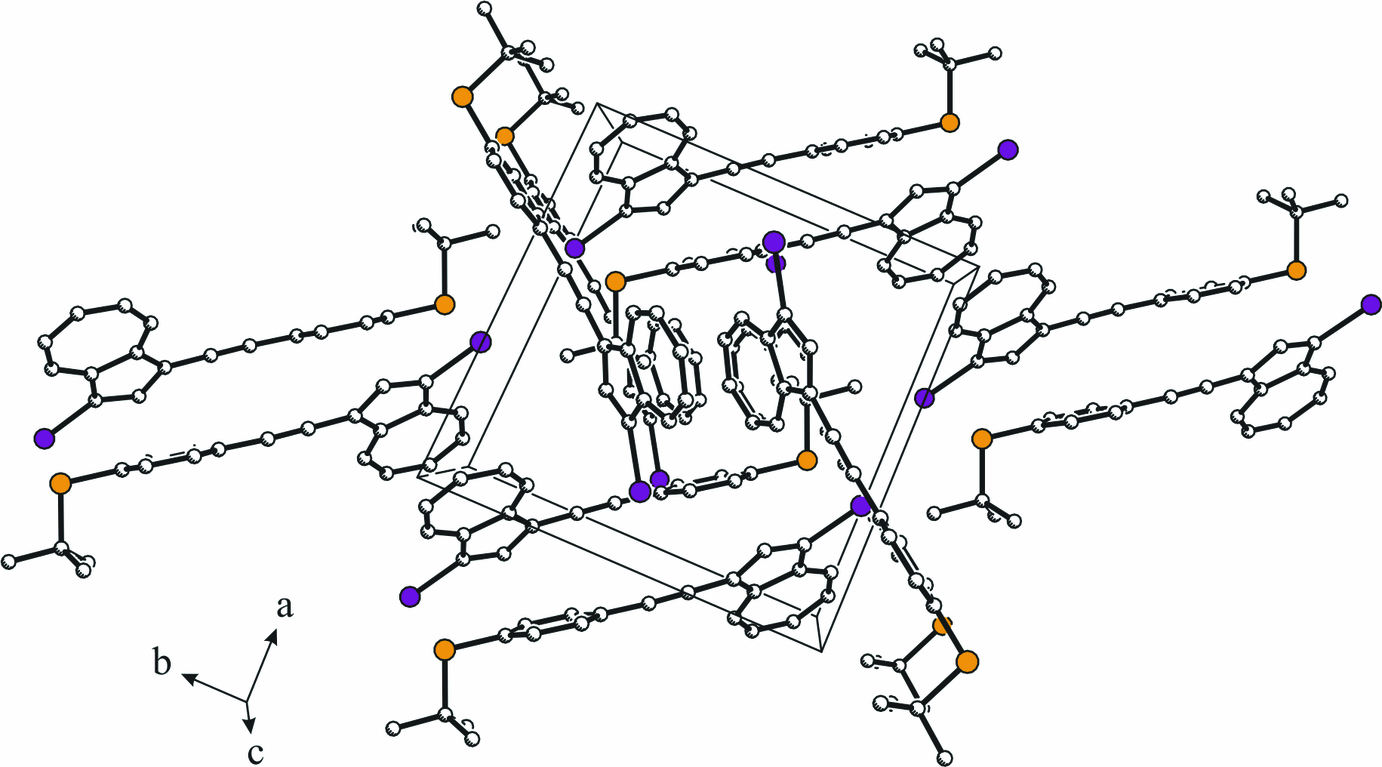

Supplement: Supplementary file 6 [file e-71-0o544-fig2.tif]
